# The Wellcome Historical Museum

**Published:** 1921-02-19

**Authors:** 


					The Wellcome Historical Museum
seni^u^ntion^ilI"8tratinff ?val and military
to this modi ^ i ' rea* War has recently ')<x7' are
? .Vs ^. . al museum. The obiects displayed . l:ngtr
3?5?t and of^rifTinS ??.rftSfr
on the lt'ifflnr '}Vrn^' a"^ photographs depicting hos-
pitals ?iiiil.nl ' first-aid posts, dressing-sta^o > thc
var^us Fr f"?"' ar!<J tr???Port of the wounded&
manvnJhr * f,;om *>noe to Egypt. Besides th??ired
from flip /?'!,.an 8ll.r^1^n' objects and appliances j
hospitals - ]'* a,"s 1,1 battleships, submarines? a j.joU
f.eRs of u" ??llCS ,?f nM>dicaI interest from the ^ i?
i intended i'< l "hole forms but the nucleus ? ^jtif
! illustrating +1 a J"-?r? ^'.uplete and extensive ex jfis-
i torieal Awr i ?."t Jocf which will form part ?L, r0 o1'
I r?rioal Medical Museum who., Bi.nce permit*. >1^
with belief
wouiuica (luring the war. a Itr- u,.
There has also l>een added to the Museum ? , to u?
.'10 Great Mace which was recently Pre*Xrfl?r
American College of Surgeons by the constil cr^^rLa^
of the British Armies. Tliis fine piece of me <l ^jr y
s|np, which was designed and carried out W ry ??ria'
Ramsden, has been placed in the TTall of S .
Museum, where it will remain as n. perni'1' ?jjp ^ a?<1
on tins side of the Atlantic to the good f''1 , ]rirrir!l
was established l>etwecn the sur<reons of ?'
dro'it .Britain during1 tlie Great War.

				

